# Plasmonic Response to Liquid–Solid Phase Transition in Individual Gallium Nanoparticles

**DOI:** 10.1021/acs.jpclett.5c02035

**Published:** 2025-08-21

**Authors:** Michal Horák, Michael Foltýn, Vojtěch Čalkovský, Vojtěch Mikerásek, Miroslav Bartošík, Jindřich Mach, Tomáš Šikola

**Affiliations:** † Central European Institute of Technology, 48274 Brno University of Technology, Purkyňova 123, Brno 612 00, Czech Republic; ‡ Faculty of Mechanical Engineering, Institute of Physical Engineering, 48274 Brno University of Technology, Technická 2, Brno 616 69, Czech Republic

## Abstract

Gallium is a phase-changing plasmonic material offering ultraviolet-to-near-infrared tunability, facile and scalable preparation, and good stability of nanoparticles. In this work, we explore the impact of the liquid-to-solid phase transition on their plasmonic properties at the single-particle level by analytical transmission electron microscopy. We observed a phase transition from liquid to β-gallium with a freezing temperature around −135 °C and a melting temperature around −20 °C. We have shown that the dipole mode of localized surface plasmon resonances can be tuned through their size from the ultraviolet to visible spectral region, while the differences in localized surface plasmon energies between liquid gallium at 25 °C and β-gallium nanoparticles at −177 °C are minor. Our results show that the performance of gallium nanoparticles is, in the case of temperature-dependent experiments, unaffected by the liquid-to-solid phase change of gallium and paves the way for suppressing the nonradiative recombination in surface-enhanced Raman spectroscopy at cryogenic temperature.

## Graphical abstract

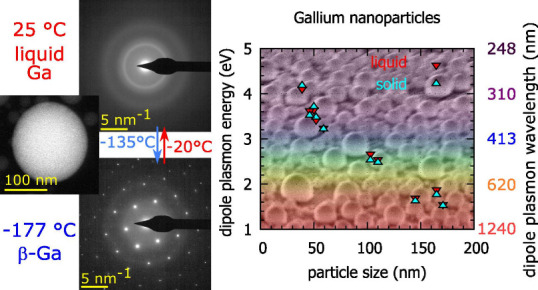

Localized surface plasmon resonances (LSPR) are collective oscillations of free electrons in metallic nanostructures coupled to the local electromagnetic field. Their characteristic feature is a strong enhancement of the electromagnetic field within the surrounding dielectric together with its confinement on the subwavelength scale, which can be utilized in numerous applications.^1–4^ The most common plasmonic metals are gold and silver. However, their performance is restricted in lower wavelengths by interband transitions. This led to the search for suitable plasmonic materials among non-noble metals. The ultraviolet and whole visible spectral range is covered by aluminum,^5^ magnesium,^6^ bismuth,^7^ silver amalgam,^8^ and gallium.^9,10^ In addition, phase-changing plasmonic materials such as vanadium dioxide^11^ or gallium^12–14^ can be utilized in specific application fields, including tunable plasmonic devices or nanoscale memories.

Bulk gallium is a nontoxic metal with a melting temperature of 29.7 °C that is acceptable to the environment.^15,16^ In addition, gallium is known to have several solid state phases that enable a variety of phase-changing systems. The liquid phase, γ phase, and δ phase of gallium has a nearly Drude-like optical response from the infrared to ultraviolet spectral region, while the α and β phases exhibit interband absorption already in the red and green spectral region.^12,13,17,18^ Fortunately, this absorption is not strong enough to completely suppress plasmonic resonances.^12^ Gallium nanoparticles can be prepared using various bottom-up fabrication techniques,^19–23^ while the low melting temperature of gallium allows low-temperature fabrication with low energy consumption. Previous studies reported plasmonic properties of gallium nanoparticles at room temperature,^9,10,24^ Ga–Ga_2_O_3_ core–shell structures,^25,26^ and gallium-based alloys.^27,28^ There are numerous applications for such nanoparticles, including biosensing platforms,^29^ surface-enhanced Raman spectroscopy applications,^30–33^ or batteries.^19,34^

In this paper, we present a temperature-dependent study of individual gallium nanoparticles by electron energy loss spectroscopy in a scanning transmission electron microscope (STEM-EELS). We perform LSPR measurements at room temperature and cryogenic temperature to address the optical response of individual gallium nanoparticles in both the liquid and the solid phase. We show a nearly identical spectral tunability of the in-plane dipole LSP mode for both phases that covers the spectral range from near-infrared to ultraviolet and correlate it with the size of gallium nanoparticles. We have grown gallium nanoparticles onto a thick silicon nitride membrane by deposition of gallium atoms using a gallium effusion cell under ultrahigh vacuum conditions (see the Methods section). The resulting nanoparticles manifest a lens-like morphology,^10^ as evidenced by the tilted view of the sample captured using a scanning electron microscopy (SEM) system (see ).

First, the sample was inspected by transmission electron microscopy (TEM). The STEM high-angle annular dark-field (HAADF) micrograph is shown in Figure 1a. The size of the nanoparticles ranges from 20 nm to 180 nm in diameter. The stability of the nanoparticles under electron beam illumination during measurement, as well as when stored on air, has been demonstrated. The long-term stability of the sample was confirmed by complementary chemical analysis using STEM energy dispersive X-ray spectroscopy (EDX), which was performed on the sample that had been stored in air for a period of one year (see ).

**Figure 1:**
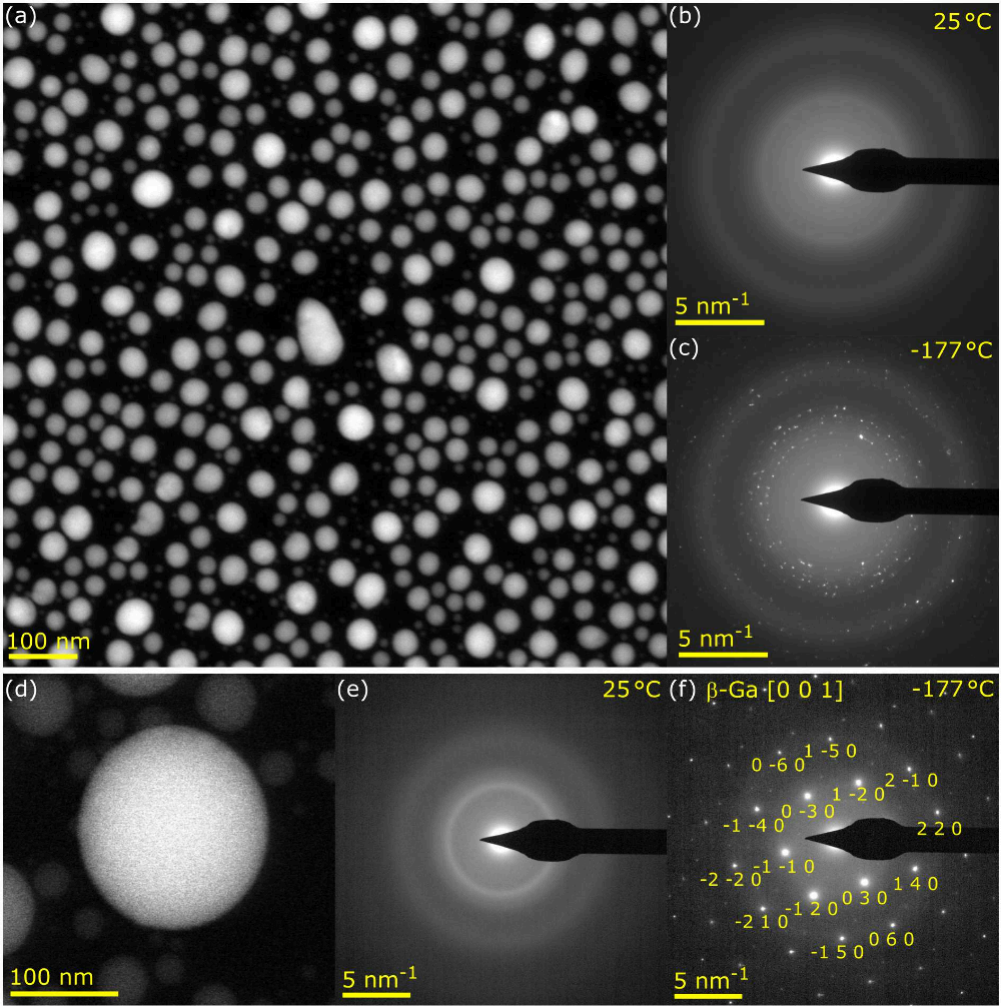
(a) STEM HAADF micrograph of a set of gallium nanoparticles on a silicon nitride membrane. (b, c) SAED of the set of gallium nanoparticles at (b) 25 °C indicating the liquid phase of gallium and (c) −177 °C proving the solid phase of gallium. (d) STEM HAADF micrograph of a single gallium nanoparticle and (e, f) SAED of this nanoparticle at 25 °C (panel (e)) denoting the liquid phase of gallium and −177 °C (panel (f)), indicating the beta phase of gallium in the [0 0 1] orientation.

Second, we explored the crystallography of the gallium nanoparticles in a temperature-dependent selective area electron diffraction (SAED) experiment. The diffraction patterns of the nanoparticles shown in Figure 1a are shown in Figure 1b for room temperature (25 °C) indicating the supercooled liquid phase of gallium and in Figure 1c for cryo temperature (−177 °C) proving the solid crystalline phase of gallium. We observed a single phase transition within the cooling from room temperature (25 °C) to cryo temperature (−177 °C) while the freezing temperature was (−135 ± 5) °C. Similarly, only one phase transition was detected within the heating back from −177 °C to 25 °C while the melting temperature was (−20 ± 10) °C. The observed transition temperatures are consistent with the previous X-ray diffraction experiment.^35^ Selected diffraction patterns recorded within this cooling–heating experiment are shown in .

Next, we performed a set of single-particle SAED experiments to determine one of the six solid phases of gallium.^13^ We tested the matching of diffraction patterns with crystallograhic models of α-gallium,^36^ β-gallium,^37^ γ-gallium,^38^ δ-gallium,^39^ gallium-II,^40^ and gallium-III^41^ using CrysTBox software.^42^ The only crystallographic phase of gallium that was in agreement was the β phase. The analysis, performed on an isolated well-oriented nanoparticle, is summarized in Figures 1d–f. Figure 1d shows the STEM HAADF micrograph of the nanoparticle with a diameter of 170 nm. The room temperature (25 °C) diffraction pattern, shown in Figure 1e, indicates the supercooled liquid phase of gallium. This finding is consistent with previous measurements reported in the literature.^10,19,35^ The cryotemperature (−177 °C) diffraction pattern, shown in Figure 1f, then indicates the β phase of gallium in the [0 0 1] orientation. This result is in agreement with the previous X-ray diffraction experiment.^35^ In addition, we note that nanoparticles smaller than 50 nm were found to crystallize into the δ phase of gallium.^19^ In contrast, the phase diagram of gallium nanoparticles introduced in ref 13 displays contradictory results, when compared with our findings and those of the aforementioned experiments.

Third, we have focused on the plasmonic properties of gallium nanoparticles that are measured by STEM-EELS at room temperature and cryotemperature to demonstrate possible differences in optical properties associated with the liquid-to-solid phase change. The processed (i.e., background subtracted and with measured counts transformed into loss probability) low loss EEL spectrum, shown in Figure 2 contains four peaks. The first two peaks correspond to the in-plane dipole mode (around 1.6 eV) and the in-plane quadrupole mode (around 2.9 eV) of LSPR, the third peak around 6.5 eV can be assigned to the surface plasmon, and the fourth peak around 14 eV corresponds to the peak of gallium volume plasmon.^10^ There are negligible differences in the LSPR peaks between the room temperature (red line) and the cryo-temperature (blue line) measurement. In contrast, the surface and volume plasmon peaks shift in energy. These shifts can be attributed to both the temperature and the phase change. The energy of the volume plasmon peak increases from 13.8 eV to 14.0 eV for the temperature change from 25 °C to −177 °C. A similar temperature-induced energy shift of the volume plasmon peak has been reported for aluminum, where the isolated temperature-related energy shift of the volume plasmon peak was used for nanoscale thermometry by EELS.^43^

**Figure 2:**
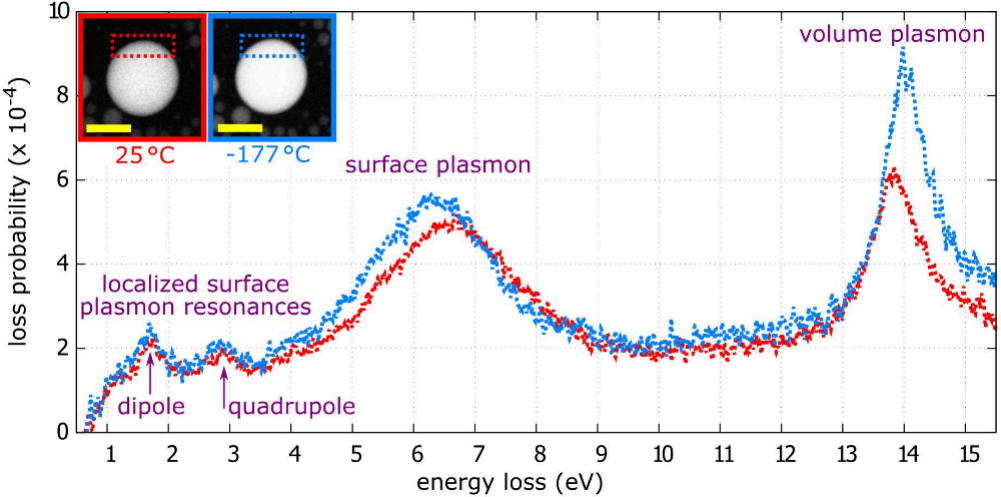
Low-loss EELS of 171-nm gallium nanoparticle recorded at its edge (integration area is marked by the rectangles) at 25 °C (red) and −177 °C (blue). The scale bar in the insets is 100 nm long. The EEL spectrum contains four peaks: the first two peaks correspond to the in-plane dipole mode (around 1.6 eV) and the in-plane quadrupole mode (around 2.9 eV) of LSPR, the third peak around 6.5 eV corresponds to the surface plasmon and the fourth peak around 14 eV corresponds to the volume plasmon.

In the following, we focus on the energy range from 1 eV to 5 eV that covers the spectral range of LSPR. Figure 3 shows the processed EEL spectra of 10 gallium nanoparticles measured at 25 °C and −177 °C. No significant differences are observed in the spectra measured at 25 °C (Figure 3a) and −177 °C (Figure 3b). The peaks corresponding to individual LSPR modes are fitted by Gaussians to determine their energy, full width at half-maximum (FWHM), which is necessary to know for Q factor evaluation, and maximum loss probability. The Q factor (quality factor), defined as the LSPR energy divided by its FWHM, describes the sharpness of a resonance. A high Q factor is indicative of a narrow resonance line width, signifying that the plasmonic structure efficiently stores energy with minimal loss. The results are summarized in Table 1. The differences in LSPR energies between liquid gallium nanoparticles at room temperature and β-gallium nanoparticles at −177 °C are minor. The energies of the dipole mode are comparable, while the largest difference is 0.14 eV for nanoparticles with diameters of 103 and 110 nm. Similarly, the energies of the quadrupole mode are comparable, with the largest difference of 0.15 eV for the 103 nm nanoparticle. Consequently, the energy shift related to the temperature-induced phase change is rather small to be utilized, for example, as a temperature sensor. However, the small change is valuable for cryogenic temperature-suppressed nonradiative recombination in surface-enhanced Raman spectroscopy.^44,45^ We note that previous theoretical calculations predicted the largest differences in LSPR energy at energies below 2 eV,^10^ where some of the Ga phases possess interband transitions.^18^ However, in this work, we have observed an energy shift of just 0.03 eV for the largest 171 nm nanoparticle with a dipole mode energy of 1.55 eV as liquid and 1.52 eV as solid, respectively. In the case of maximum loss probabilities and Q factors, no clear trend that could be assigned to the phase change has been observed because the values are affected by systematic uncertainties, such as instrumental broadening, arising from the measurement principles and conditions themselves. The Q factors of the dipole mode are around 2 for most nanoparticles, which is consistent with the previous experiment.^10^

**Figure 3:**
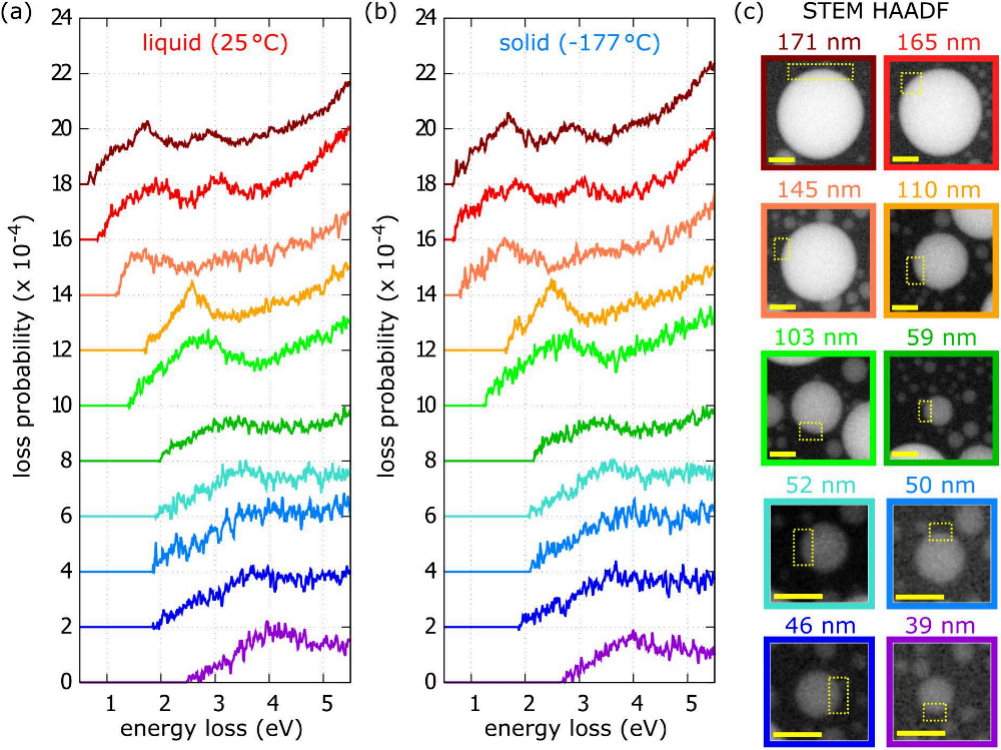
(a, b) Low-loss EELS of 10 gallium nanoparticles with the diameter from 39 nm to 171 nm recorded at (a) 25 °C and (b) −177 °C. (c) STEM HAADF micrographs of these nanoparticles. The EEL spectra were integrated over the same areas at nanoparticles edges (marked by yellow rectangles) at both temperatures. The scale bars in panel (c) are 50 nm long.

**Table 1:** Plasmonic Properties of Liquid and Solid Gallium Nanoparticles

		Dipole Mode			Quadrupole Mode		
size (nm)		energy (eV)	loss prob.	Q factor	energy (eV)	loss prob.	Q factor
171	liq.	1.55	1.22 × 10^–5^	1.6	2.93	1.48 × 10^–5^	1.2
	sol.	1.52	1.82 × 10^–5^	1.6	2.80	1.57 × 10^–5^	1.7
165	liq.	1.88	1.82 × 10^–5^	1.2	3.04	1.44 × 10^–5^	5.3
	sol.	1.76	1.87 × 10^–5^	1.1	3.11	1.43 × 10^–5^	3.9
145	liq.	1.69	1.05 × 10^–5^	2.4	3.45	1.21 × 10^–5^	1.2
	sol.	1.63	1.53 × 10^–5^	1.6	3.33	1.31 × 10^–5^	1.6
110	liq.	2.54	1.84 × 10^–5^	3.1	3.65	0.61 × 10^–5^	3.2
	sol.	2.48	1.92 × 10^–5^	2.8	3.69	0.47 × 10^–5^	4.5
103	liq.	2.67	2.15 × 10^–5^	1.8	4.35	0.35 × 10^–5^	4.5
	sol.	2.53	1.86 × 10^–5^	1.6	4.20	0.23 × 10^–5^	6.1
59	liq.	3.24	1.22 × 10^–5^	1.7			
	sol.	3.22	0.97 × 10^–5^	2.6			
52	liq.	3.42	0.71 × 10^–5^	4.5			
	sol.	3.48	0.74 × 10^–5^	4.2			
50	liq.	3.64	0.77 × 10^–5^	2.7			
	sol.	3.71	0.54 × 10^–5^	2.7			
46	liq.	3.62	1.23 × 10^–5^	2.6			
	sol.	3.52	0.99 × 10^–5^	2.2			
39	liq.	4.11	0.75 × 10^–5^	4.8			
	sol.	4.18	0.66 × 10^–5^	3.1			

Figure 4 shows the energy of the dipole and quadrupole LSPR mode in a set of gallium nanoparticles in a liquid phase (red and dark-red) and β phase (blue and dark-blue), as a function of their diameter. The size of the nanoparticles was determined from STEM-HAADF micrographs (shown in Figure 3c). The uncertainty in particle size corresponds to the size of two pixels in the image and is 4 nm. The error bars of the energies include a standard error of the Gaussian fit to the experimental spectrum and a systematic error, primarily related to the FWHM of the zero-loss peak (ZLP), estimated to be 0.15 eV. In the case of liquid gallium nanoparticles, the energy of the dipole LSPR mode increases from 1.55 eV for the largest nanoparticle (171 nm in diameter) to 4.11 eV for the smallest one (39 nm in diameter) with decreasing particle diameter. Similarly, in the case of solid gallium nanoparticles, the energy of the dipole LSPR mode increases from 1.52 eV for the largest nanoparticle to 4.18 eV for the smallest one. The dipole LSPR mode of the gallium nanoparticle in both phases (liquid and β) can be tuned from the ultraviolet spectral region, represented by the nanoparticle with a diameter of 39 nm with dipole resonances around 300 nm in wavelength, to the red end of the visible spectral region, represented by the nanoparticle with a diameter of 171 nm with dipole resonances around 810 nm in wavelength. Our results are in agreement with previous studies.^9,10^ The quadrupole LSPR mode, detected in particles larger than 100 nm then covers the ultraviolet and blue part of the visible spectral range represented with the 103 nm nanoparticle with quadrupole resonances around 290 nm in wavelength and the 171 nm nanoparticle with quadrupole resonances around 430 nm in wavelength.

**Figure 4:**
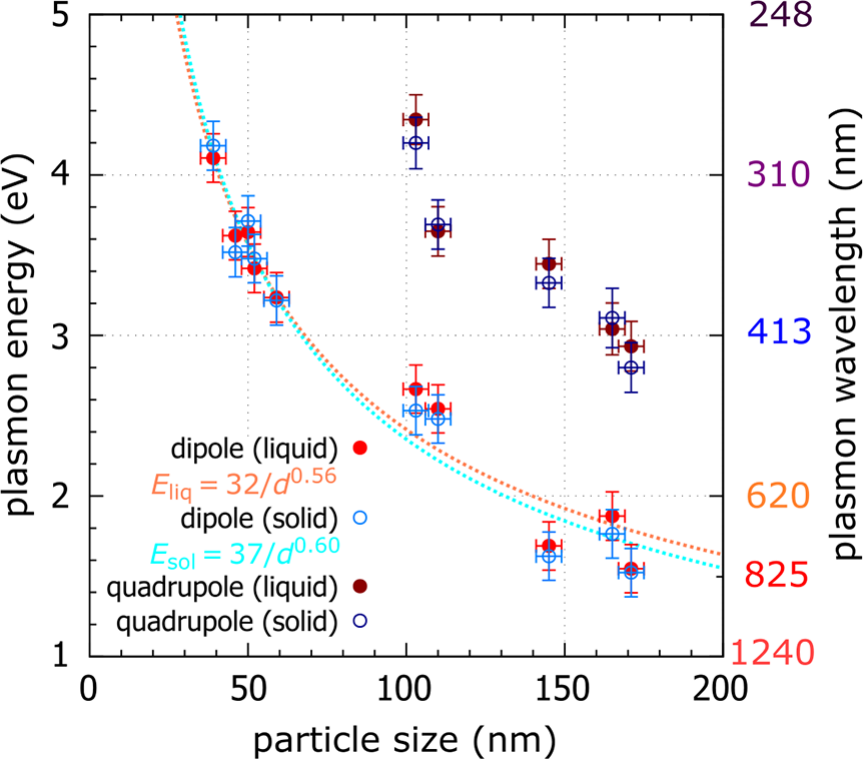
Energy of the dipole and quadrupole mode of localized surface plasmon resonances in a set of gallium nanoparticles in a liquid (red and dark-red) and solid (blue and dark-blue) phase covering the spectral region from the ultraviolet across the visible to the near-infrared. Experimental data obtained from the EELS measurement are further fitted by empirical power laws *E*(*d*) = *A*/*d* ^*n*^, with the dipole mode energy *E* expressed in electron volts and the particle diameter *d* in nanometers, reading *E*_liq_(*d*) = 32/*d* ^0.56^ for the liquid and *E*_sol_(*d*) = 37/*d* ^0.60^ for the solid phase.

The experimental dependencies in Figure 4 can be reasonably well approximated by the empirical power law *E*(*d*) = *A*/*d* ^*n*^, with the dipole mode energy *E* expressed in electron volts, the particle diameter *d* in nanometers, and dimensionless parameters *A* and *n*.^10^ The empirical parameters *A* and *n* determined from the least-squares method are listed in Table 2. The experimental dependence for solid appears to be slightly steeper than for liquid nanoparticles, but the parameter *A* (32 ± 6 for liquid, compared to 37 ± 7 for solid gallium) and the exponent *n* (0.56 ± 0.05 for liquid, compared to 0.60 ± 0.05 for solid gallium) are equal in both cases within the uncertainty. Similarly, the values of *A* and *n* for liquid nanoparticles are equal to the experimental values reported in ref 10 within the uncertainty. The theoretical values of *A* and *n* reported in ref 10 differ slightly, suggesting a minor inaccuracy in determining the shape, dimensions, or dielectric function of the particles utilized in the numerical model.

**Table 2:** Comparison of Parameters of the Empirical Power Law *E*(*d*) = *A*/*d* ^*n*^

dataset	*A*	*n*
liquid Ga (measured at 25 °C)	32 ± 6	0.56 ± 0.05
solid Ga (measured at −177 °C)	37 ± 7	0.60 ± 0.05
liquid Ga experiment^a^	24 ± 3	0.48 ± 0.03
liquid Ga simulation^a^	11.7 ± 1.4	0.33 ± 0.03
solid Ga simulation^a^	11.1 ± 0.3	0.299 ± 0.006

a Data taken from ref 10.

In conclusion, we have explored the plasmonic properties of gallium nanoparticles at room temperature and cryogenic temperature −177 °C on a single particle level. Gallium nanoparticles with diameters in the range from 20 nm to 180 nm were grown directly on the silicon nitride membrane by deposition of gallium atoms using a gallium effusion cell under ultrahigh vacuum conditions. We observed a single phase transition within the cooling from room temperature to −177 °C while the freezing temperature was around −135 °C. Similarly, only one phase transition was detected within the heating back from −177 °C to 25 °C while the melting temperature was around −20 °C. The solid phase was identified using selective area electron diffraction as β-gallium. Plasmonic properties were measured by STEM-EELS. We have shown that their dipole mode can be tuned via their size from the ultraviolet to visible spectral region, while the differences in LSPR energies between liquid gallium nanoparticles at room temperature and β-gallium nanoparticles at −177 °C are minor. Consequently, the energy shift related to the temperature-induced phase change is rather small to be utilized, for example, as a temperature sensor. With regard to potential applications, previous works reported the use of gallium nanoparticles to enhance luminescence or as a biosensing platform. Our results show that, in the case of temperature-dependent experiments the performance of gallium nanoparticles should be unaffected by the liquid-to-solid phase change of gallium and paves the way for suppressing the nonradiative recombination in surface-enhanced Raman spectroscopy by cryogenic temperature.

## Methods

Samples with gallium nanoparticles were prepared by direct deposition of gallium atoms on a silicon nitride membrane (50 nm thick) using a gallium effusion cell developed in house under UHV conditions as introduced in ref 10. The size of the resulting nanoparticles is influenced by the temperature of the substrate, the gallium flux, and the time of deposition. In this work, gallium was deposited on the silicon nitride membrane heated to (320 ± 10) °C using a calibrated pyrolytic boron nitride heating element. The deposition time was 3 h with a gallium flux density of 7.2 × 10^12^ atoms s^–1^ cm^–2^ and the chamber pressure of 3.8 × 10^–8^ Pa.

TEM analysis was performed with a TEM FEI Titan equipped with a GIF Quantum spectrometer operated in TEM mode at 300 keV for imaging and diffraction, and in scanning monochromated regime at 120 keV for electron energy loss spectroscopy (EELS). The sample was inserted into a Gatan cryo-holder which allows the sample to be cooled by liquid nitrogen, resulting in temperatures available in the range from −177 °C to +100 °C. In the case of the STEM EELS measurement of LSPR, the beam current was 0.1 nA, the full width at half-maximum of the zero-loss peak (ZLP) was 0.15 eV, the convergence semiangle was 10 mrad, the collection semiangle was 11.4 mrad, and the dispersion of the spectrometer was 0.01 eV/pixel. The acquisition time was adjusted to exploit the maximum intensity range of the CCD camera, avoiding overexposure. These parameters were selected to acquire the EELS signal with the highest signal-to-background ratio.^46^ To reduce the noise in the LSPR signal, we integrated the EEL spectra over the rectangular areas at the nanoparticle edges where the generation of LSPR and thus the loss probability is significant. They were further divided by the integral intensity of the ZLP to transform the measured counts to a quantity proportional to the loss probability (reffered as the loss probability), background (membrane) subtracted and fitted by Gaussians.

## Supplementary Material





## Notes

Tilted view of lens-shaped gallium nanoparticles on a silicon nitride membrane; complementary chemical analysis of gallium nanoparticles stored in air; selected TEM micrographs, and corresponding diffraction patterns from the cooling–heating experiment with a set of gallium nanoparticles (PDF)
Transparent Peer Review report available (PDF)
