# Supplementary material for: Plasmonic Response to Liquid–Solid Phase Transition in Individual Gallium Nanoparticles
Source: J Phys Chem Lett. 2025 Aug 21;16(35):8891–6. doi: 10.1021/acs.jpclett.5c02035 (PMC12415875; doi:10.1021/acs.jpclett.5c02035)
Supplement: Supplementary file 1 [file jz5c02035_si_001.pdf]

Supplementary Information: Plasmonic Response to Liquid-Solid Phase Transition in Individual Gallium Nanoparticles

Michal Horák,[*,†] Michael Foltýn,[†] Vojtěch Čalkovský,[†,‡] Vojtěch Mikerásek,[†,‡] Miroslav Bartošík,[†,‡] Jindřich Mach,[†,‡] and Tomáš Šikola[†,‡]

†*Brno University of Technology, Central European Institute of Technology, Purkyňova 123, Brno, 612 00, Czech Republic*

‡*Brno University of Technology, Faculty of Mechanical Engineering, Institute of Physical Engineering, Technická 2, Brno, 616 69, Czech Republic*

E-mail: michal.horak2@ceitec.vutbr.cz

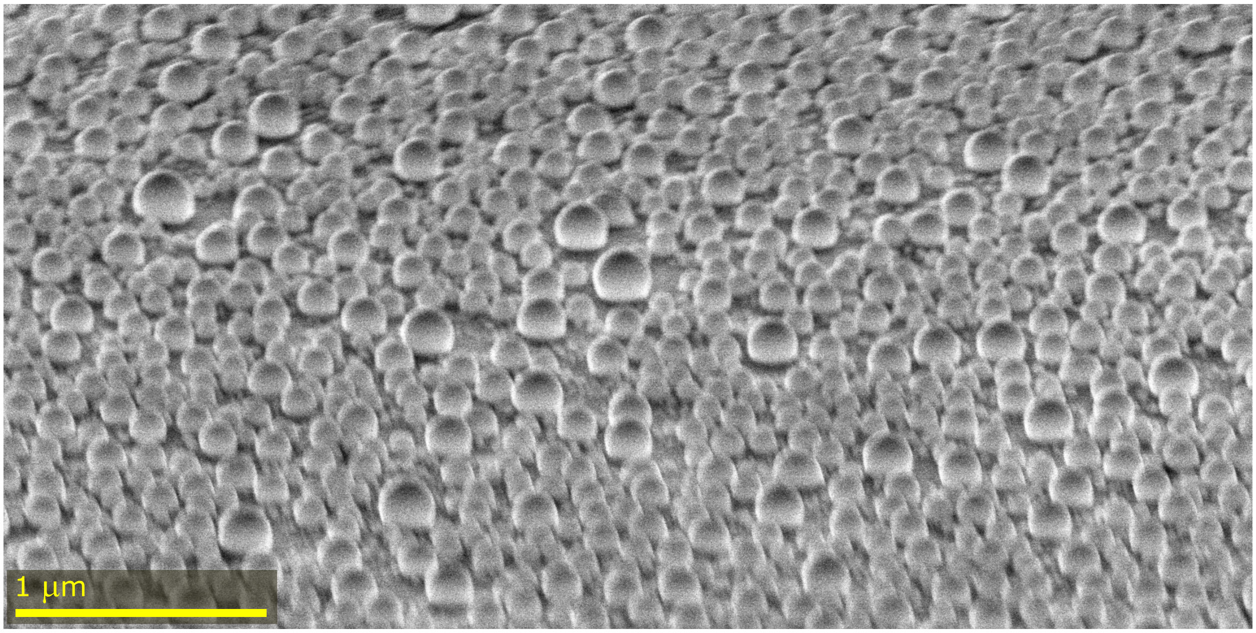

Figure S1: Tilted view of lens-shaped gallium nanoparticles on a silicon nitride membrane captured using a scanning electron microscope. The tilt of the sample is 60.4° and secondary electrons signal is used.

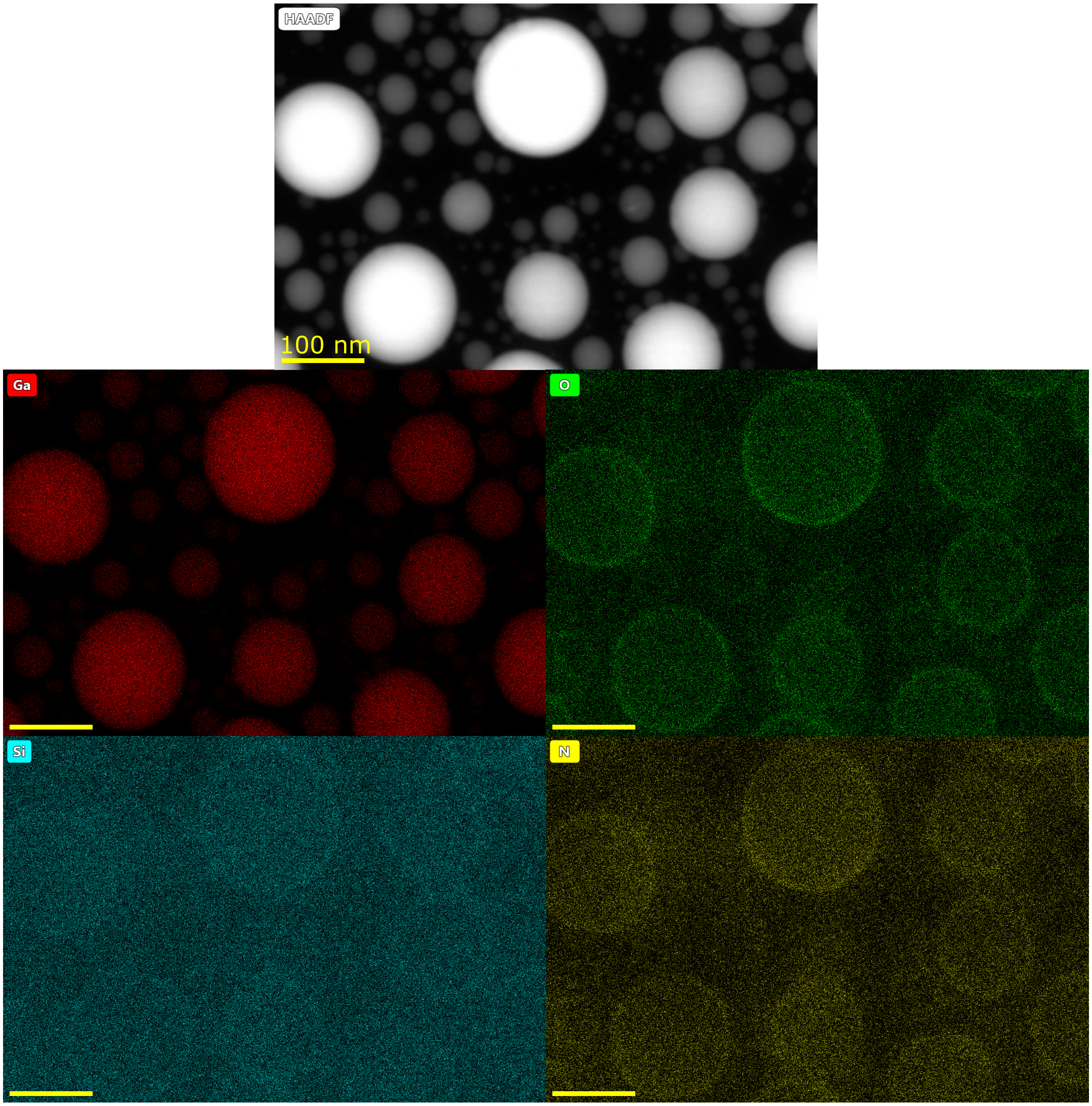

Figure S2: Complementary chemical analysis using STEM EDX performed on gallium nanoparticles stored in air for a period of one year confirming the long-term stability of the sample. STEM HAADF micrograph followed by net intensity elemental maps of Ga (red), O (green), Si (cyan), and N (yellow). The native oxide layer on gallium nanoparticles is just a few nanometers thick. The Si and N signal comes from the silicon nitride membrane.

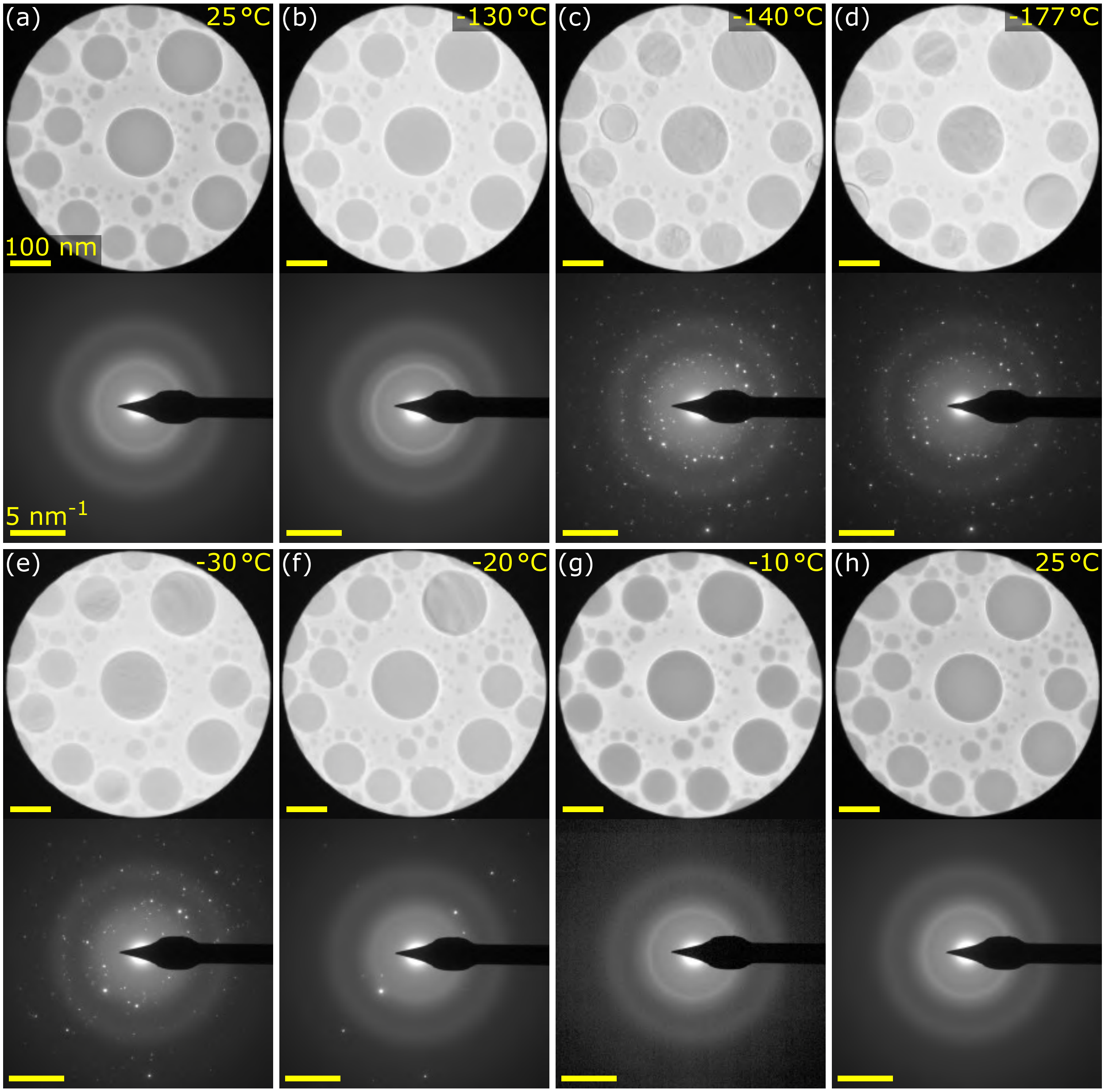

Figure S3: Cooling-heating experiment with a set of gallium nanoparticles. Selected TEM micrographs and corresponding diffraction patterns are shown for the temperature of 25 °C (a), −130 °C (b), −140 °C (c), −177 °C (d), −30 °C (e), −20 °C (f), −10 °C (g), and 25 °C (h). A single phase transition is observed within the cooling as well as within the heating. The freezing temperature is around −135 °C and the melting temperature is around −20 °C.
