# Supplementary material for: Plasmonic Response to Liquid–Solid Phase Transition in Individual Gallium Nanoparticles
Source: J Phys Chem Lett. 2025 Aug 21;16(35):8891–6. doi: 10.1021/acs.jpclett.5c02035 (PMC12415875; doi:10.1021/acs.jpclett.5c02035)
Supplement: Supplementary file 2 [file jz5c02035_si_002.pdf]

jz-2025-020355.R1

Name: Peer Review Information for "Plasmonic Response to Liquid-Solid Phase Transition in Individual Gallium Nanoparticles"

First Round of Reviewer Comments

Reviewer: 1

Comments to the Author

-In Methods section, it is said that the temperature of the Ga deposition process is 320 ºC. Is it from the substrate or from the crucible? How did you check and control the temperature? Are they any error bars produced by the setup?

-Page 4, line 38. Instead of -20ºC I recommend to write -15 ± 5 ºC since your measurements were taken at -20ºC with some solid pattern and -15ºC with liquid pattern as they are shown in Fig. S1. I also recommend to express the freezing temperature to be -135 ± 5 ºC for the same reason.

-I do not understand last paragraph of page 5 and first paragraph of page 6. So, I understood that you have found a gallium phase (β) that you did not expect. Reference 13 visualized other phases for the same material and nanoparticle size. Can you explain why those data are contradictory?

-How is the shape of the Ga nanoparticles? They typically form an hemispherical shape almost in any substrates. Did you confirm it in silicon nitride? If it is that the case, there should be two modes of resonances: in-plane and out-of-plane resonances as explained in a lot of your references (22th for example). What of those are you measuring? I guess the in-plane because of the low energy of the dipole modes. You should include this discussion for the possible readership.

-Page 7, first paragraph. It is quite interesting that you have found a difference of the bulk plasmon energy from liquid to solid state. Is it related to the dielectric function of each one? By looking at the Drude-like function of each case, the bulk plasma frequency could be anticipated, right?

-Page 7, line 56. You should explain what is Q factor and why it should be considered important. Only plasmonics readership is familiar with this parameter.

-Page 8, first paragraph. I agree that LSPR energy different is quite small but I am not sure if Q factor change between liquid and solid state is negligible. In fact, in page 9 first paragraph you admit that there are systematic uncertainties that provoke Q factor differences. So, you should specify the sentence “The diferences between liquid gallium nanoparticles at room temperature and β gallium nanoparticles at 177 ºC are minor” to be only applied to the LSPR energies.

-Page 3, line 33, you stated that β gallium phase has interband absorption in the green and red spectral region. But in your EELS measurements in Fig. 3, nanoparticles with sizes of 110-160 nm are showing LSPRs in that regions at low temperature where they are β phase. In fact, the Q factor values are around 1 to 3. Interband transitions are supposed to suppress plasmonic resonances. This seems contradictory. Can you explain it?

Author's Response to Peer Review Comments:

Ing. Michal Horák, Ph.D.
Brno University of Technology, CEITEC
Purkyňova 123, 612 00 Brno, Czech Republic
michal.horak2@ceitec.vutbr.cz

August 1, 2025

**Response to the Decision Letter**

Dear Editor,

we thank you for handling our manuscript and the Reviewer for the comments, which we thoroughly utilized to improve the manuscript, and we are glad to submit the Revised Manuscript article entitled "Plasmonic Response to Liquid-Solid Phase Transition in Individual Gallium Nanoparticles" by Michal Horák, Michael Foltýn, Vojtěch Čalkovský, Vojtěch Mikerásek, Miroslav Bartošík, Jindřich Mach, and Tomáš Šikola for consideration for publication in *The Journal of Physical Chemistry Letters*.

The full response to the Reviewer's comments follows at the end of this document. Further, the manuscript PDF with all changes made is uploaded as the Supplementary material for Review only, too.

This manuscript is not under consideration for publication elsewhere.

We thank you for the care that you take of our manuscript.

Sincerely,
Michal Horák
on behalf of all Authors

**Response to the Editorial and Reviewer's comments**

We thank the Reviewer for very useful comments, which we thoroughly utilized to improve the manuscript. A point-by-point response to the Editorial and Reviewer's comments follows. The Reviewer's report is in blue, our comments in black, and the corresponding action in green.

## Editorial comments

1. Please include author names, title of article, name of repository (section name, if provided), submission date, DOI or URL (accessed YYYY-MM-DD) for the following incomplete preprint references: 7.

We completed the bibliography information in the .bib file.

## Reviewer 1

Recommendation: This paper may be publishable, but major revision is needed; I would like to be invited to review any future revision.

We thank Reviewer 1 for the evaluation of our manuscript.

Comments:

-In Methods section, it is said that the temperature of the Ga deposition process is 320 ºC. Is it from the substrate or from the crucible? How did you check and control the temperature? Are they any error bars produced by the setup?

We thank the reviewer for a very good comment. It is the temperature of the substrate. The substrate is heated by a pyrolytic boron nitride (PBN) heating element that contains graphite wires, which heat the PBN as electric current is applied. First, a calibration curve of the substrate temperature as a function of electric current had to be determined. While increasing the electric current (including the time for temperature stabilization), the temperature is measured with a K-type thermocouple coupled to the PBN heating element with the substrate in ultra-high vacuum conditions. The calibration is individual to each PBN heating element and remains stable unless the experimental conditions are significantly changed. The temperature uncertainty is estimated to be less than ±10 °C.

The corresponding text in Methods was updated to: *"…The size of the resulting nanoparticles is influenced by the temperature of the substrate, the gallium flux, and the time of deposition. In this work, gallium was deposited on the silicon nitride membrane heated to (320 ± 10) °C using a calibrated pyrolytic boron nitride heating element. The deposition time was 3 h with a gallium flux density of…"*

-Page 4, line 38. Instead of -20ºC I recommend to write -15 ± 5 ºC since your measurements were taken at -20ºC with some solid pattern and -15ºC with liquid pattern as they are shown in Fig. S1. I also recommend to express the freezing temperature to be -135 ± 5 ºC for the same reason.

We thank the reviewer for a very good comment. We agree that using a temperature interval would be better than using the word "around". Consequently, we rewrote the freezing temperature to (–135 ± 5) °C, as the –130 °C measurement indicates the liquid and the –140 °C measurement indicates the solid phase. The melting

temperature was rewritten to (–20 ± 10) °C, as the –30 °C measurement indicates full solid, the –20 °C measurement indicates partial solid and partial liquid and the –10 °C measurement indicates full liquid phase.

The corresponding text was updated to: *"...while the freezing temperature was (–135 ± 5) °C."* and *"...while the melting temperature was (–20 ± 10) °C."*

-I do not understand last paragraph of page 5 and first paragraph of page 6. So, I understood that you have found a gallium phase (β) that you did not expect. Reference 13 visualized other phases for the same material and nanoparticle size. Can you explain why those data are contradictory?

Our results (experiment, selective area electron diffraction of nanoparticles with diameters >50 nm) indicate the β-gallium phase.

An overview of the literature gives the following:

- A study by A. Cicco [10.1103/PhysRevLett.81.2942] (experiment, X-ray diffraction of nanoparticles) indicates the β-gallium phase, too.
- A study by M. Yarema et al. [10.1021/ja506712d] (experiment, X-ray diffraction and selective area electron diffraction of nanoparticles with diameters <50 nm) indicates the δ-gallium phase. This result is not contradictory, as the size of the nanoparticles is different.
- A study by Y. Gutiérrez et al. [10.1515/nanoph-2020-0314] (Reference 13) introduces in Figure 1 the phase diagram of bulk Ga, Ga nanoparticles as a function of nanoparticle size, and a scheme of various Ga thin films that can be obtained as a function of the deposition temperature, reconstructed from several referenced sources. It also includes an ε-gallium, for which the crystalographic model is not available. In the case of Ga nanoparticles, the cited source is another study by Y. Gutiérrez et al. [10.1002/adom.201900307] in which no phase diagram is presented. Consequently, the origin of the phase diagram for Ga nanoparticles in the study by Y. Gutiérrez et al. [10.1515/nanoph-2020-0314] is rather mysterious. This phase diagram predicts the γ- and δ-gallium for nanoparticles with diameters between 50 and 300 nm, depending on the temperature. It also does not reflect the fact that gallium nanoparticles are at room temperature in the form of a supercooled liquid. Consequently, the main question is the correctness of the introduced phase diagram for Ga nanoparticles.

Consequently, our results are in agreement with A. Cicco [10.1103/PhysRevLett.81.2942] and not contradictory to M. Yarema et al. [10.1021/ja506712d]. On the other hand, the phase diagram of Ga nanoparticles by Y. Gutiérrez et al. [10.1515/nanoph-2020-0314] is contradictory to all three experimental works discussed above (i.e., our results, results by A. Cicco [10.1103/PhysRevLett.81.2942], and results by M. Yarema et al. [10.1021/ja506712d]) as it predicts γ-gallium (300 K to 200 K) and δ-gallium (below 200 K) for nanoparticles with diameters ranging from 50 nm to 300 nm, and γ-gallium (300 K to 185 K) and ε-gallium (below 185 K) for smaller nanoparticles. Additionally, it does not reflect the fact that gallium nanoparticles are at room temperature in the form of a supercooled liquid. Consequently, the phase diagram for Ga nanoparticles introduced by Y. Gutiérrez et al. [10.1515/nanoph-2020-0314] (Reference 13) in Figure 1 seems to be incorrect.

The corresponding text was simplified to: *"...This result is in agreement with a previous X-ray diffraction experiment [10.1103/PhysRevLett.81.2942]. In addition, we note that nanoparticles smaller than 50 nm were found to crystallize into the δ phase of gallium [10.1021/ja506712d]. In contrast, the phase diagram of gallium nanoparticles introduced in Ref. [10.1515/nanoph-2020-0314] displays contradictory results when compared with our findings and those of the aforementioned experiments."*

-How is the shape of the Ga nanoparticles? They typically form an hemispherical shape almost in any substrates. Did you confirm it in silicon nitride? If it is that the case, there should be two modes of resonances: in-plane and out-of-plane resonances as explained in a lot of your references (22th for example). What of those are you measuring? I guess the in-plane because of the low energy of the dipole modes. You should include this discussion for the possible readership.

The hemispherical or the lentil shape of the Ga nanoparticles was confirmed in the case of silicon nitride, too. This was discussed in our previous publication [10.1021/acs.jpclett.3c00094]. We agree that this information might be missing in this paper, and we include it in the revised manuscript.

The following text was added: *"The resulting nanoparticles manifest a lens-like morphology [10.1021/acs.jpclett.3c00094], as evidenced by the tilted view of the sample captured using a scanning electron microscope (see Figure S1)."*

An additional figure was added to the Supplementary Information (Figure S1).

Regarding the in-plane and out-of-plane resonances, we measure the in-plane ones. We agree that this information is missing in the paper, and we include it in the revised manuscript.

The corresponding text was updated to: *"…in-plane dipole LSP mode…"* and *"The first two peaks correspond to the in-plane dipole mode (around 1.6 eV) and the in-plane quadrupole mode (around 2.9 eV) of LSPR…"*.

-Page 7, first paragraph. It is quite interesting that you have found a difference of the bulk plasmon energy from liquid to solid state. Is it related to the dielectric function of each one? By looking at the Drude-like function of each case, the bulk plasma frequency could be anticipated, right?

The shift of volume plasmon energy is a well-known temperature-induced effect that takes into account the volume change of the material (i.e., the thermal expansion) and the consequent change of dielectric properties. It can be used for nanoscale thermometry by EELS [10.1016/j.ultramic.2019.112881]. In our case, the situation is more complicated as we have two temperatures and two different phases of gallium. In the case of a Drude-like dielectric function, the bulk plasma frequency could be anticipated. However, β-gallium has intraband transitions and is not considered a Drude-like metal [10.1002/adom.201900307]. As the focus of this manuscript is on LSPR, we find additional experiments related to volume plasmon to be out of the scope of this manuscript.

The corresponding (unchanged) text in the manuscript is:

*"There are negligible differences in the LSPR peaks between the room temperature (red line) and the cryo-temperature (blue line) measurement. In contrast, the surface and volume plasmon peaks shift in energy. These shifts can be attributed to both the temperature and the phase change. The energy of the volume plasmon peak increases from 13.8 eV to 14.0 eV for the temperature change from 25 °C to –177 °C. A similar temperature-induced energy shift of the volume plasmon peak has been reported for aluminum, where the isolated temperature-related energy shift of the volume plasmon peak was used for nanoscale thermometry by EELS [10.1016/j.ultramic.2019.112881]."*

No change in the manuscript.

-Page 7, line 56. You should explain what is Q factor and why it should be considered important. Only plasmonics readership is familiar with this parameter.

In plasmonics, the Q factor (quality factor) describes the sharpness of a localized surface plasmon resonance or surface plasmon resonance. It is typically defined as the ratio of the resonance's central wavelength to its full-width half-maximum. A high Q factor indicates a narrow resonance linewidth, meaning the plasmonic structure efficiently stores energy with minimal loss.

The following text was added: *"The Q factor (quality factor), defined as the LSPR energy divided by its FWHM, describes the sharpness of a resonance. A high Q factor is indicative of a narrow resonance linewidth, signifying that the plasmonic structure efficiently stores energy with minimal loss."*

-Page 8, first paragraph. I agree that LSPR energy different is quite small but I am not sure if Q factor change between liquid and solid state is negligible. In fact, in page 9 first paragraph you admit that there are systematic uncertainties that provoke Q factor differences. So, you should specify the sentence "The diferences between liquid gallium nanoparticles at room temperature and β gallium nanoparticles at 177 ºC are minor" to be only applied to the LSPR energies.

We thank the reviewer for a very good comment. We agree that it is better to connect the minor differences just to the LSPR energies, as the Q factors, as well as the peak intensities, might be affected by systematic uncertainties arising from the experiment.

The corresponding text was updated to: *"...the differences in LSPR energies between liquid gallium nanoparticles at room temperature and β-gallium nanoparticles at –177 °C are minor."*

-Page 3, line 33, you stated that β gallium phase has interband absorption in the green and red spectral region. But in your EELS measurements in Fig. 3, nanoparticles with sizes of 110-160 nm are showing LSPRs in that regions at low temperature where they are β phase. In fact, the Q factor values are around 1 to 3. Interband transitions are supposed to suppress plasmonic resonances. This seems contradictory. Can you explain it?

Interband transitions are indeed supposed to suppress plasmonic resonances. An obvious example is gold with strong interband transitions above 2.2 eV (below 550 nm in wavelength). However, in the case of β-gallium, the interband transitions are not strong enough to completely suppress plasmonic resonances. For example, the study by Y. Gutiérrez et al. [10.1002/adom.201900307] introduces in Figure 5 the longitudinal dipole LSPR for a β-gallium nanorod at 1.7 eV and for an α-gallium nanorod at 2.2 eV, while both α- and β-gallium have intraband transitions in the considered spectral region. The imaginary part of the dielectric function of β-gallium does not exceed the value of ~50, and the imaginary part of the refractive index of β-gallium does not exceed the value of ~5 [10.1515/nanoph-2020-0314]. Further, we note that this dielectric function is calculated and not experimentally verified. Consequently, we expect a small broadening of the plasmon peak as the LSPRs are supposed to be "lossy". This is a common case of any non-perfect plasmonic material with a non-negligible damping. The Q factor values around 1 to 3 are indeed not high at all and reflect the instrumental broadening of LSPR peaks, as well as the material damping.

We added the following sentence (in green) into the introduction: *"The liquid phase, γ phase, and δ phase of gallium has a nearly Drude-like optical response from the infrared to ultraviolet spectral region, while the α and β phases exhibit interband absorption already in the red and green spectral region [10.1002/adom.201900307, 10.1515/nanoph-2020-0314, 10.1038/s41598-017-05985-8, 10.1364/ome.9.004050]. Fortunately, this absorption is not strong enough to completely suppress plasmonic resonances [10.1002/adom.201900307]."*

**CEITEC – Central European Institute of Technology**
Brno University of Technology
Purkyňova 123, 612 00 Brno, Czech Republic
www.ceitec.eu

jz-2025-020355.R2

Name: Peer Review Information for "Plasmonic Response to Liquid-Solid Phase Transition in Individual Gallium Nanoparticles"

Second Round of Reviewer Comments

Reviewer: 1

Comments to the Author

The authors have responded all my questions and suggestions. I recommend the publication of this paper including the proposed changes by the authors.

Author's Response to Peer Review Comments:

Dear Editor,

we thank you for handling our manuscript. According to the editorial comment

"1. Headers: Remove the section heading(s) throughout the body of the manuscript (you can leave Methods, Abstract, and TOC Graphic headings)."

we have removed the two subsection headings that were in the previous version in the Methods section. No other change was made.

Sincerely,

Michal Horák

on behalf of all Authors
